# Effects of MN_4_-Type Coordination Structure in Metallophthalocyanine for Bio-Inspired Oxidative Desulfurization Performance

**DOI:** 10.3390/molecules27030904

**Published:** 2022-01-28

**Authors:** Gai Zhang, Yufan Zhang, Amin Tan, Yan Yang, Min Tian

**Affiliations:** School of Materials Science and Chemical Engineering, Xi’an Technological University, Xi’an 710021, China; zhangyufan@st.xatu.edu.cn (Y.Z.); Tanamin@st.xatu.edu.cn (A.T.); Yangyan@xatu.edu.cn (Y.Y.)

**Keywords:** metallophthalocyanine, MN_4_-type coordination structure, desulfurization, O_2_

## Abstract

Oxidative desulfurization (ODS) is the promising new method for super deep desulfurization of fuel oil. The oxidative desulfurization performance of the metal-N_4_-chelates metallophthalocyanines (MPcs) is related to the chemical properties of conjugate structures and the central metal ions. Herein, a biomimetic catalytic system composed of metallophthalocyanines (MPcR_4_, M = Mn(II), Fe(II), Co(II), Ni(II), Cu(II), Zn(II); R = -H, -COOH, -NO_2_, -NH_2_) and molecular O_2_ was performed to study the influence of MN_4_-type coordination structure in metallophthalocyanines for the degradation of dibenzothiophene (DBT) in model oil containing n-octane. The results reveal that the conjugate structures and the center metal ions of metallophthalocyanines played key roles in oxidative desulfurization performance. The inductive effect of different R substituents strongly affected the electron cloud distribution of the conjugate structures and the catalytic performance. Moreover, the catalytic activity of MPcs, which is related to the d electronic configuration and ligand-field effects, does not sequentially increase with the increase in the d electron number of central metal ions.

## 1. Introduction

With the aggravation of environmental pollution problems, the removal of sulfur compounds in vehicle fuel has become an important research focus. The sulfur oxyacids (SO_X_) produced by fuels combustion remained a major source of air pollution and acid rain. Thiophene and its derivatives are the main sulfur compounds [1,2,3]. Oxidative desulfurization technology is one of the most effective super deep desulfurization methods for thiophene and its derivatives [4,5]. Many alternative oxidative desulfurization technologies have been attempted based on the semiconductor materials, graphene, carbonnanotubes, metal macrocycles and their appropriate combinations. Specially, many reports have pointed out that metal macrocycles have broad prospects in the field of petroleum refining as the catalyst for the removal of sulfur-containing compounds [6,7,8].

**Figure 1:**
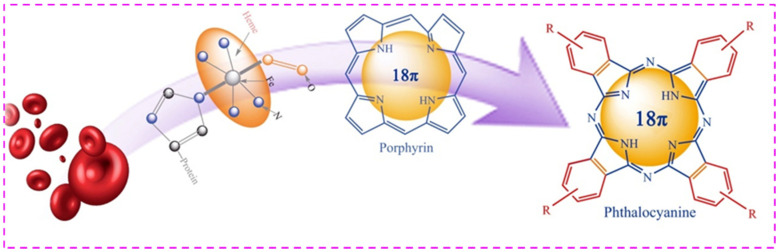
Biomimetic oxygen carrier function of phthalocyanine.

Metal-N_4_-chelate compound is one of numerous metal macrocycles. Metalloporphyrin (MPs) and metallophthalocyanine (MPcs) with metal-N_4_-chelate structure have an 18 π electrons conjugated structure and have the function of biomimetic oxygen carrier resembling hemeproteins in the human body [9] (Figure 1). Compared with MPs, a variety of substituents can modify with the periphery of the benzene ring of phthalocyanines, and different substituents can make MPcs have different chemical structures and properties [10,11,12]. The catalytic activity of the metal-N_4_-chelate macrocycles is affected by the chemical properties of the ligand field and the central metal ion. Owing to the π electron conjugated structure of the metal-N_4_-chelates, delocalization effect and weakly bonding properties, metal-N_4_-chelate compounds are more easily oxidized and reduced. A phthalocyanine ring is a cyclic rotaene chromophore with 18 π electrons. The p–π conjugation of substituent and phthalocyanine ring influences the electron cloud density distribution of the system and catalytic activity. For MPcs with strong ligand-field effects, the chemical structure of the phthalocyanine, the radius and the d-filling of the metal center ions are the main factors that influence catalytic activity [13]. Furthermore, the existence of electronegative substituents extremely reinforced the catalytic activity of the phthalocyanines. Their catalytic activity is reinforced by the numerous electron-withdrawing substituents on the periphery of metal Pcs [14]. The results stated clearly that the revision of the main MPc_s_ skeleton enhanced catalytic performance and encouraged us to design a phthalocyanine compound with different substituents. In this paper, the metallophthalocyanines ZnPc, ZnPc(COOH)_4_, ZnPc(NH_2_)_4_ and ZnPc(NO_2_)_4_, which have the same central metal ion and different electron-withdrawing substituents, were prepared to study the influence of chemical structure on oxidative desulfurization. At the same time, the phthalocyanine molecules themselves do not have catalytic oxidation activity. Metallophthalocyanines have excellent catalytic performance when the phthalocyanine molecules are coordinated with the metal ions to form phthalocyanine complexes. Therefore, the type of central metal ions is also the key factor affecting the catalytic activity of phthalocyanine complexes.

Additionally, H_2_O_2_ is the most common oxidant in catalytic oxidative desulfurization technologies. However, the reduction product H_2_O of H_2_O_2_ causes a two-phase mass transfer problem in the reaction system, and further damages oil quality [15,16]. In addition, the expensive H_2_O_2_ results in the catalysts having a significant cost in large-scale application. Molecular oxygen (O_2_) would be a desired oxidant for ODS due to the great advantages, such as being inexpensive, obtainable, and environmentally friendly [17,18]. However, the triplet state molecular oxygen (^3^O_2_) makes it difficult to react with an organic compound of a singlet state at room temperature; that is, spin and symmetry are prohibited. Phthalocyanine is a P-type semiconductor. The phthalocyanine complex is excited by visible light to form the triplet excited state (^3^[MPc]*). The triplet excited state phthalocyanine(^3^[MPc]*) interacts with the triplet ground state oxygen molecule (^3^O_2_) to transfer energy and generate singlet state oxygen molecules (^1^O_2_) with strong activity. Singlet state oxygen molecules oxidize and decompose organic compounds [19]. Therefore, molecular oxygen is an ideal oxidant for the catalytic oxidative desulfurization system of the phthalocyanine complex. Zhou Xinrui [20] et al. studied the catalytic oxidation of thiophene in alkanes by O_2_/FePc(NO_4_)_4_ system. The results showed that the phthalocyanine complex can effectively activate oxygen molecules and achieve deep desulfurization.

**Figure 2:**
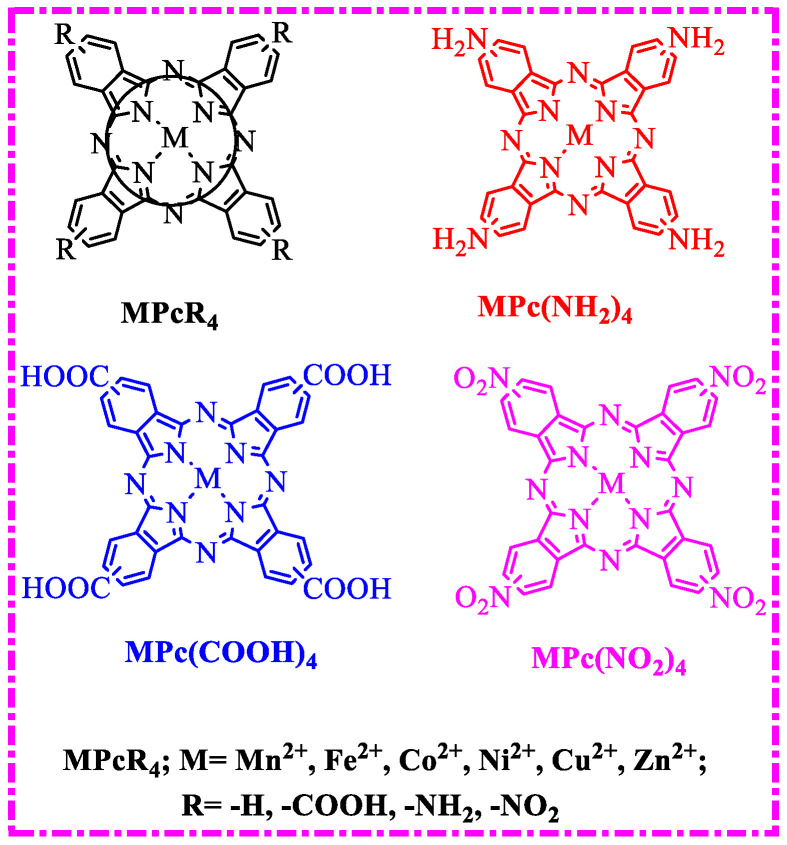
The structure of metal phthalocyanine.

In this paper, the phthalocyanine complexes MPcR_4_ (MPcR_4_, M = Mn^2+^, Fe^2+^, Co^2+^, Ni^2+^, Cu^2+^, Zn^2+^; R = -H, -COOH, -NH_2_, -NO_2_, Figure 2) were selected as desulfurization catalysts, the O_2_ molecule was used as the oxidant, dibenzothiophene (DBT) simulated sulfur-containing pollutants, and a biomimetic catalytic system was performed to study the influence of MN_4_-type coordination structure in metallophthalocyanines for the degradation of dibenzothiophene(DBT). Based on the results, a “double active site” of phthalocyanine complexes for catalytic oxidation desulfurization was proposed.

## 2. Results and Discussion

### 2.1. Structural Characterization of Phthalocyanine Complexes

**Figure 3:**
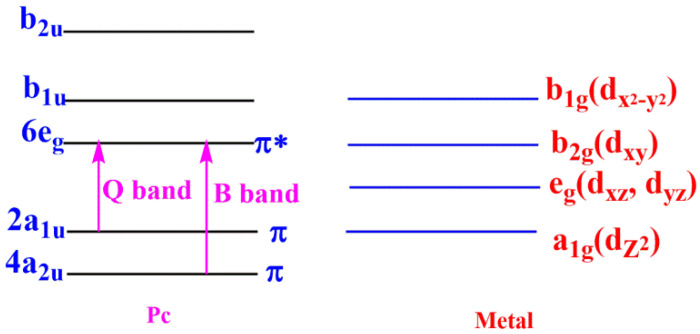
The diagram of molecular orbital of MPc.

**Figure 4:**
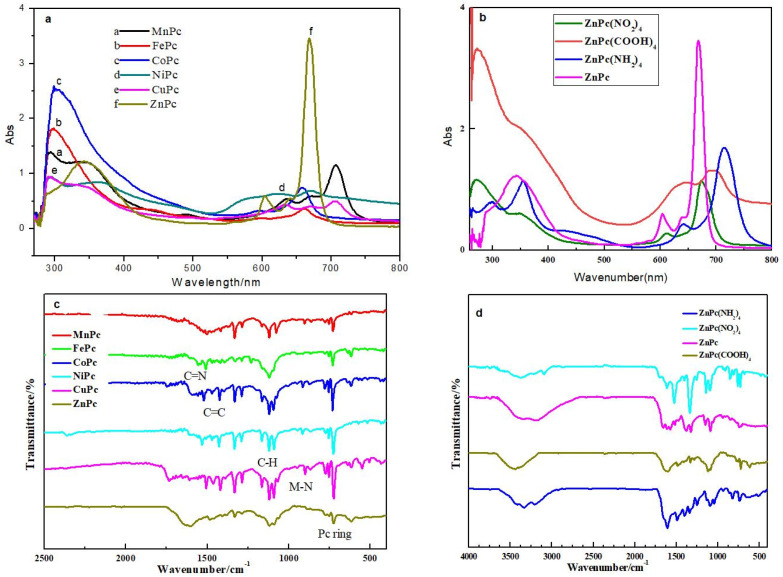
UV-Vis and FTIR spectra of MPcR_4_ compounds. (**a**,**b**): UV-Vis spectra of MPcR_4_; (**c**,**d**): FTIR spectra of MPcR_4_.

The metallophthalocyanines MPcR_4_ were characterized by FT-IR, UV-Vis spectrum analysis and elemental analysis. The results of quantum mechanical calculations showed that the UV-Vis spectrum of the phthalocyanine complexes have two absorption bands—the Q band and the B band—which belong to the π electron transition of the phthalocyanine ring (Figure 3). The spectral properties of the phthalocyanine complex were further studied by UV-Vis spectrum, as shown in Figure 4a,b. The Q band is located at 600–700 nm with the energy of about 3.8 eV, which belongs to the electronic transition of 2a_1u_ (LUMO) → 6_eg_ (HOMO). The B-band is located in the wavelength range of 250–350 nm with the energy of 1.8 eV, which belongs to the electronic transition of 4a_2u_ → 6_eg_ [8]. The absorption peak of the Q band is the characteristic absorption band of phthalocyanine ring. In addition, when the substituents were introduced on the conjugated system of the phthalocyanine complex, the red shifts occurred in the B band and Q band. The phthalocyanine ring is a cyclic rotaene chromophore with 18 π electrons. The p–π conjugation effect between the substituent and the phthalocyanine ring strongly affected the distribution of the electron cloud density and the position of Q band. The Q-band shifted to the near-infrared region because the energy difference ΔE between LUMO (Highest Occupied Molecular Orbital) and HOMO (Lowest Unoccupied Molecular Orbital) decreased with the orderly increase in the electron cloud density for the ZnPc, ZnPc (NO_2_)_4_, ZnPc (COOH)_4_ and ZnPc (NH_2_)_4_ systems. Additionally, the Q-band intensity of a series of MPc complexes was obviously enhanced. This was probably due to the increase in d electrons in the central metal ions (Mn^2+^, Fe^2+^, Co^2+^, Ni^2+^, Cu^2+^, Zn^2+^) and the decrease in energy difference ΔE between LUMO and HOMO. Furthermore, the intensity of Q band decreased, obviously due to aggregation caused by hydrogen bonds and intermolecular forces [21,22]. The results indicate that the aggregation effect broadened the UV absorption peaks.

The FT-IR spectra were recorded to further investigate the structure of the MPcR_4_ compounds (Figure 4c,d). The characteristic absorption peak at 3342 cm^−1^ assigned –OH stretching vibration. The absorption peak in the range of 887–920 cm^−1^ belonged to the M-N bond formed by the central metal ion and the nitrogen atom on the pyrrole ring. The absorption peaks at 1680–1550 cm^−1^ are attributed to the stretching vibration peak of C=C and C=N bond on the phthalocyanine ring. The absorptions in 1000–1200 cm^−1^ were assigned to the characteristic absorption of the C-H stretching vibration. Noteworthily, the appearance of strong absorption peak at 750–720cm^−1^ indicated the formation of phthalocyanine conjugated macrocycles. In addition, 3324, 3203cm^−1^ absorption peak belonged to the NH_2_ group [23,24]. The stretching vibration peaks of N-O bond were located at 1522, 1336 cm^−1^.

### 2.2. Catalytic Oxidation Activity of Phthalocyanine Complexes (MPcR_4_)

#### 2.2.1. Effect of Central Metal Ion on Catalytic Oxidation Desulfurization Activity of Phthalocyanine

**Figure 5:**
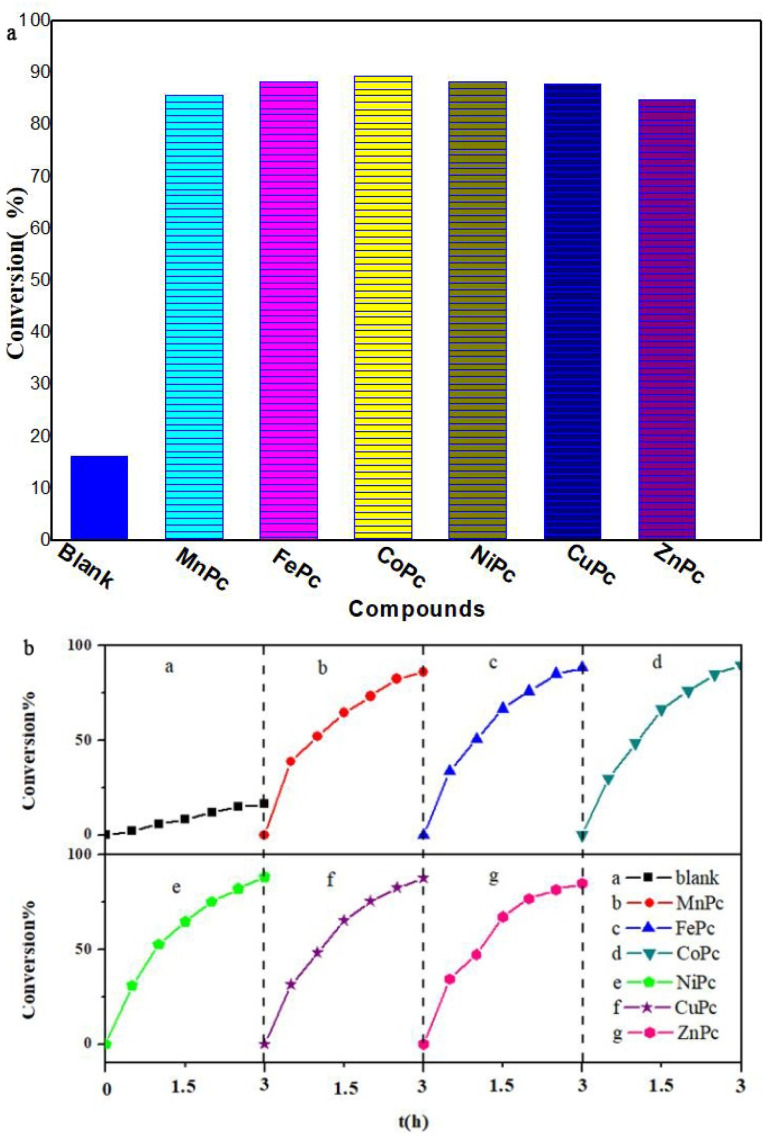
The influence of center metal ion of phthalocyanine. (**a**) desulfurization activities of phthalocyanine; (**b**) desulfurization activities at different time.

The desulfurization activities of phthalocyanine complexes with different center metal ions are in order of CoPc > FePc > NiPc > CuPc > MnPc > ZnPc (Figure 5). The experimental results indicate that the catalytic activity of phthalocyanine complexes does not increase in an orderly way with the increase in the d-electron number of the central metal ions. The results show that the d-electron configuration of the central metal ion is the main factor affecting the catalytic oxidation desulfurization activity of the phthalocyanine complexes.

The catalytic activity is related to the electron delivery ability of central metal ions. The electron delivery ability of central metal ion M(II) can be obtained by molecular orbital theory. The quantum chemical theoretical calculation method INDO/S was used to study the frontier molecular orbital characteristics of the MPc molecule, and the results are shown in Table 1 [25,26]. The d orbital contribution of metal ions in the highest occupied molecular orbital (HOMO) is in order of Co(II) > Fe(II) > Ni(II) > Cu(II) > Mn(II) > Zn(II), which is according with the catalytic oxidative desulfurization activity. Combining the catalytic activity, it can be concluded that π orbit (Fe^2+^, Mn^2+^) has more electron delivery capacity than σ orbit (Co^2+^, Ni^2+^, Cu^2+^). In the case of the same orbit type, the higher the HOMO level energy is according to the greater d orbital contribution of metal ion, the stronger the electron donating ability and the higher the catalytic activity. In addition, Zn(II) ion has the worst electron donating ability because Zn(II) ion has a fully stable structure of d^10^. Considering the coordination effect of HOMO orbital type (σ, π) and metal ion d orbit contribution, the catalytic activity of phthalocyanine complexes does not sequentially increase with the increase in d electron number of central metal ions [27,28].

#### 2.2.2. Effect of Conjugation Structure on Catalytic Oxidative Desulfurization Activity of Phthalocyanine

**Figure 6:**
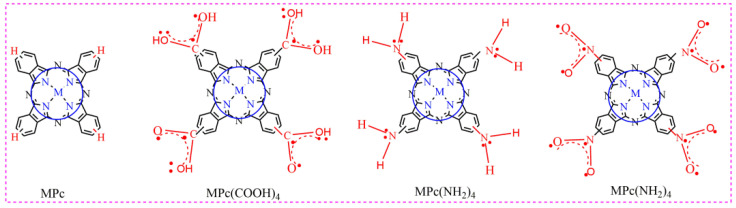
Conjugate effect of phthalocyanine structures.

**Figure 7:**
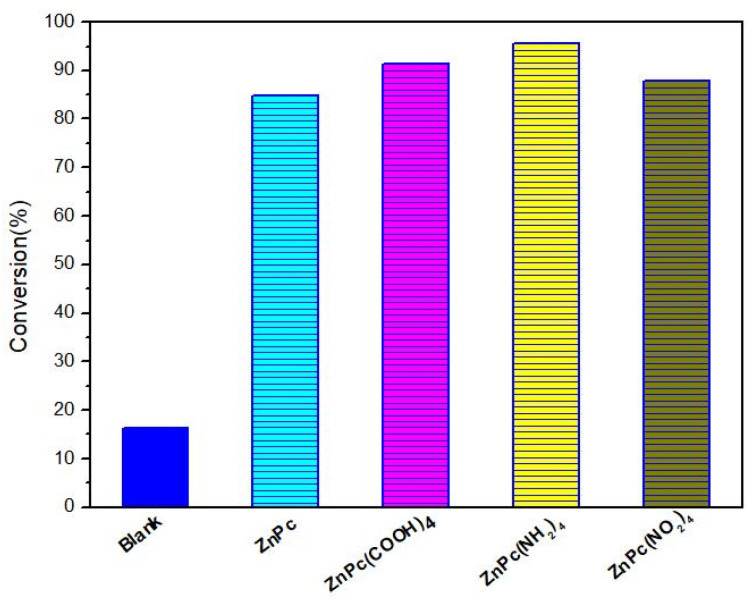
The influence of conjugate effect of phthalocyanine structures.

In addition, the nature of the substituents on the phthalocyanine ring strongly affects the catalytic oxidation desulfurization activity of the MN_4_-type conjugation system (Figure 6). The desulfurization activity is in order of ZnPc < ZnPc(NO_2_)_4_ < ZnPc(COOH)_4_ < ZnPc(NH_2_)_4_. The activity of the different substituted phthalocyanine complexes is shown in Figure 7. The results show that inductive effect and p–π conjugation effect of four R substituents are another main factor affecting the electron cloud density distribution of metal phthalocyanine and its catalytic performance. The phthalocyanine ring is a cyclic rotaene chromophore with 18 π electrons. Due to the delocalized and weakly bound character of π electron clouds, the conjugated compounds with relatively high π electron density can be oxidized and reduced relatively easily [29,30]. Considering the same central metals, the π electron density of MPcR_4_ is higher than that of MPcs because of the p–π conjugate effects of four R substituents (Figure 6). The p–π conjugate effects are benefits for the activation process of O_2_. The electron cloud density of ZnPc < ZnPc(NO_2_)_4_ < ZnPc(COOH)_4_ < ZnPc(NH_2_)_4_ system increases sequentially with the increase in inductive effect and p–π conjugation effect. The higher electron cloud density is conducive to transfer an electron from phthalocyanine to the oxygen molecule, and the catalytic oxidative desulfurization activity of phthalocyanine complexes increased sequentially.

#### 2.2.3. Effect of MN_4_-Type Coordination Structure on Catalytic Oxidation Desulfurization Activity of Phthalocyanine

**Figure 8:**
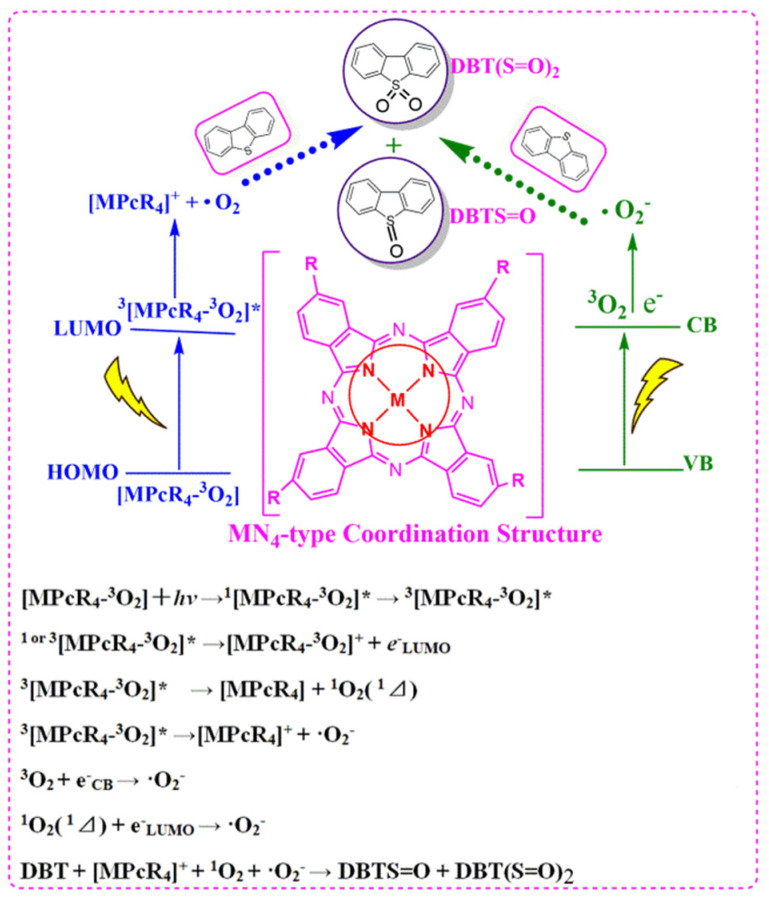
Photocatalytic degradation mechanism of MPcR_4_.

The catalytic oxidative desulfurization activity of phthalocyanine complexes was studied taking an O_2_ molecule as oxidant and dibenzothiophene as simulated sulfur pollutant. The maximum removal rate of dibenzothiophene reached 96.46% after reacting for 180 min at 60 °C. The reaction mechanism for oxidative desulfurization is shown in Figure 8. Under natural light irradiation, the singlet phthalocyanine complexes [MPcR_4_-^3^O_2_] were excited to form triplet excited state ^3^[MPcR_4_-^3^O_2_]*; ^3^[MPcR_4_-^3^O_2_]* triplet excited state phthalocyanine interacts with triplet ground state oxygen molecule ^3^O_2_ to conduct energy transfer and generate singlet oxygen molecule ^1^O_2_. The active intermediates ^*^O_2_-MPcR_4_^+^ formed strong activity oxidants [MPcR_4_]^+^ and O_2_, which converted DBT to sulfuric acid radical ion and sulphone. The quantum yield of the photocatalytic redox process increased as a result of the additional formation of oxidants [MPcR_4_]^+^ and O_2_ [16].

It was found that the catalytic activity of *O_2_-MPcR_4_^+^ is related to the molecular frontier orbital (HOMO and LUMO) properties. The molecular frontier orbital properties are determined by the electron delivery ability of the central ion and the electron cloud distribution of the conjugated system of phthalocyanine ring. The results indicate that the M^2+^ and PcR_4_ rings are both active centers. The molecular orbital theory further indicated that the properties of the frontier molecular orbitals (HOMO and LUMO) have an important influence in the reaction properties of the molecules. In the same system, the ability of donating electrons increased with the higher HOMO level energy. The ability of accepting electrons increased with the lower LUMO level energy. The catalytic activity of the metal-N_4_-chelates macrocycles is affected by the chemical properties of the ligand effect and the central metal ion.

**Figure 9:**
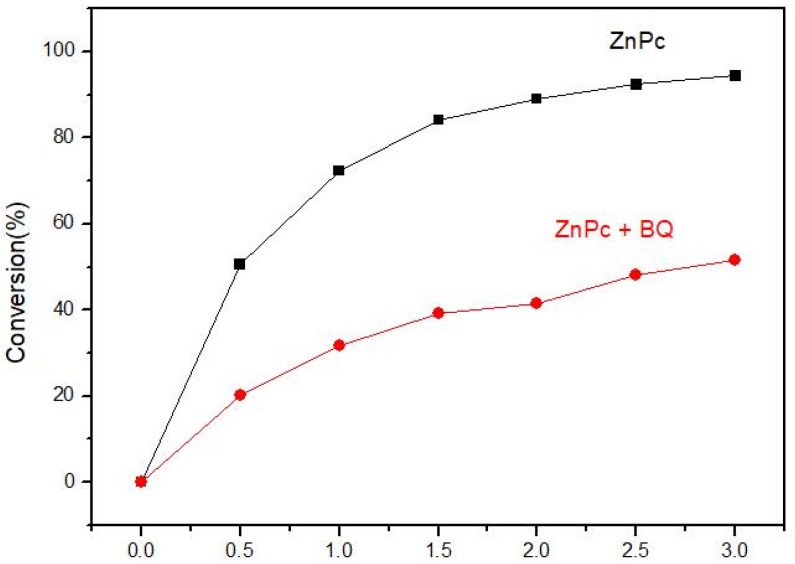
•O_2_^−^ Radical trapping experiments.

**Figure 10:**
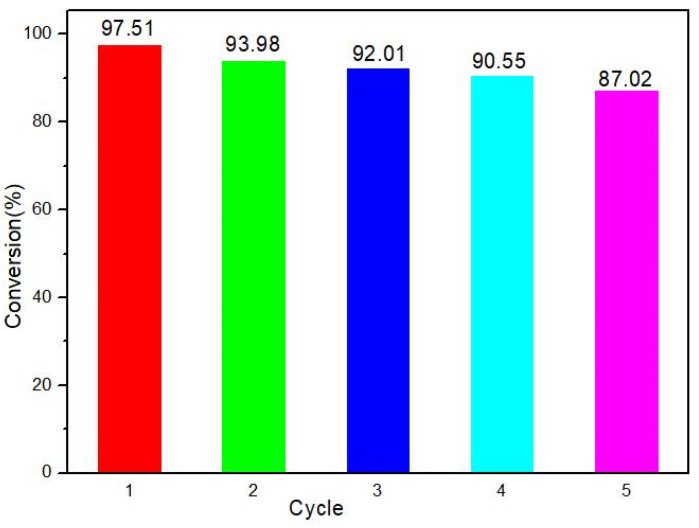
The results of stability test for the catalysts ZnPc.

Radical trapping experiments were designed to reveal the active oxygen species •O_2_^−^ on the photocatalysis process through using appropriate quenchers p-benzoquinone(BQ). The obtained results are shown in Figure 9. The dramatic decline from 94.42% to 51.61% of DBT removal is achieved with the addition of 1mM BQ. The results suggest that •O_2_ is the main active species for DBT degradation under visible light irradiation. Additionally, photostability of the catalysts is a major property for their practical application. DBT degradation was performed to test the reusability of MPc. The reaction was cooled to room temperature before being filtered off. After being separated, the catalysts were washed with distilled water for 3 times and dried at 100 °C under vacuum. The obtained results are shown in Figure 10. Although there is a decrease in the degradation ratio for each photocatalyst, more than 93.98% of dibenzothiophene can be degraded in the second circle. To further investigate the reusability of MPcs, DBT degradation was performed five times. The catalysts of ZnPc could be used at least five times with only a 10% change. The results indicate that the MPcs compounds processed high stability.

## 3. Experimental Part

### 3.1. Experimental Reagents and Instruments

Phthalic anhydride and 1,2,4-benzoic anhydride (Aladdin Chemical Reagent Co., Ltd., Shanghai, China), M(COOH)_2_, Sinopharm Chemical Reagent Co., Ltd., Shanghai, China), urea (CO(NH_2_)_2_, Tianjin Kemer Chemical Reagent Co., Ltd., Tianjin, China), dibenzothiophene(DBT) (Chengdu Kailong Chemical Reagent Co., Ltd., Chengdu, China), and all other reagents were analytical reagent grade and were used without further purification.

An elemental analyzer was used (Elementar Vario EL III, PE). The IR spectra were recorded on a Germany Bruker Equinox55 spectrometer. The UV-vis absorbance was recorded on a UV-Visible spectrophotometer (UV-2550, Shimadzu, Shanghai, China) using a quartz cell with a path length of 10 mm at room temperature. The dibenzothiophene (DBT) content was determined using an Agilent GC 6890 (Shanghai, China) with FPD detector.

### 3.2. Synthesis of Metallophthalocyanine

Metallophthalocyanine was prepared according to the method in the literature [13]. The target MPcR_4_ compounds were prepared by taking phthalic anhydride with different R substituents(R = -H, -COOH, -NH_2_, -NO_2_) and the corresponding metal salts (M = Mn^2+^, Fe^2+^, Co^2+^, Ni^2+^, Cu^2+^, Zn^2+^) as the raw materials; a mixture of 6.0000 g of urea, 0.2500 g of NH_4_Cl, and 0.1200 g of (NH_4_)_2_Mo_2_O_7_ was further added into a 100 mL three-necked flask and heated at 140 °C for 0.5 h with magnetic stirrer and reflux condenser, and then kept at 220 °C for 6 h under ambient air conditions. The by-products were washed with water, followed by 6 mol·L^−1^ hydrochloric acid for several times. Then the purification of blue solid (green solid) was achieved by refluxing with 150 mL of acetone and trichloromethane about 12 h.

Mn(II)Pc—1.1524 g (40.61%)Yield; green solid; m.p. >300 °C; IR(KBr) *ν*_max_/cm^−1^: 1617(*ν*_C=N_); 914(*ν*_M-N_); 1382, 1091, 731 (*ν*_Pc_); UV-Vis(DMF) *λ*_max_/nm: 267, 617, 719; Anal. Cald. for C_32_H_16_N_8_Mn: C, 67.73; H, 2.84; N, 19.45; Found: C, 67.25; H, 2.31; N, 20.18.

Fe(II)Pc—1.3562 g, (47.79%)Yield; blue solid; m.p. >300 °C; IR(KBr) *ν*_max_/cm^−1^: 1640(*ν*_C=N_); 903(*ν*_M-N_); 1380, 1080, 731 (*ν*_Pc_); UV-Vis(DMF) *λ*_max_/nm: 272, 662; Anal. Cald. for C_32_H_16_N_8_Fe: C, 67.62; H, 2.84; N, 19.71; Found: C, 67.33; H, 2.64; N, 19.56.

Co(II)Pc—1.2892 g, (45.39%)Yield; blue solid; m.p. >300 °C; IR(KBr) *ν*_max_/cm^−1^: 1637(*ν*_C=N_); 903(*ν*_M-N_); 1384, 1120, 730 (*ν*_Pc_); UV-Vis(DMF) *λ*_max_/nm: 331, 666; Anal. Cald. for C_32_H_16_N_8_Co: C, 66.27; H, 2.83; N, 19.73; Found: C, 66.83; H, 2.78; N, 19.43.

Ni(II)Pc—1.1299 g, (39.76%)Yield; green solid; m.p. >300 °C; IR(KBr) *ν*_max_/cm^−1^: 1635(*ν*_C=N_); 916(*ν*_M-N_); 1380, 1091, 725 (*ν*_Pc_); UV-Vis(DMF) *λ*_max_/nm: 267, 363, 670; Anal. Cald. for C_32_H_16_N_8_Ni: C, 67.28; H, 2.82; N, 19.62; Found: C, 66.45; H, 2.43; N, 19.90.

Cu(II)Pc—1.3832 g, (48.62%)Yield; blue solid; m.p. >300 °C; IR(KBr) *ν*_max_/cm^−1^: 1637(*ν*_C=N_); 898(*ν*_M-N_); 1384, 1091, 727 (*ν*_Pc_); UV-Vis(DMF) *λ*_max_/nm: 344,668; Anal. Cald. for C_32_H_16_N_8_Cu: C, 66.72; H, 2.80; N, 19.26; Found: C, 66.47; H, 2.36; N, 19.55.

Zn(II)Pc—1.1776 g, (41.52%)Yield; green solid; m.p. >300 °C; IR(KBr) *ν*_max_/cm^−1^: 1635(*ν*_C=N_); 887(*ν*_M-N_); 1384, 1087, 727 (*ν*_Pc_); UV-Vis(DMF) *λ*_max_/nm: 341, 670; Anal. Cald. for C_32_H_16_N_8_Zn: C, 67.63; H, 2.84; N, 19.72; Found: C, 67.01; H, 2.24; N, 19.26.

Zn(II)Pc(NH_2_)_4_—1.6608 g, (51.67%)Yield; blue solid; m.p. >300 °C; IR(KBr) *ν*_max_/cm^−1^: 3346, 3203(*ν*_NH2_); 1614, 830(*ν*_N-H_); 927(*ν*_M-N_); 1386, 1092, 737 (*ν*_Pc_); UV-Vis(DMF) *λ*_max_/nm: 355, 715; Anal. Cald. for C_36_H_20_N_12_Zn: C, 57.56; H, 2.18; N, 22.22; Found: C, 57.01; H, 2.23; N, 21.36.

Zn(II)Pc(COOH)_4_—0.3976 g, (23.94%)Yield; green solid; m.p. >300 °C; IR(KBr) *ν*_max_/cm^−1^: 3342(*ν*_O-H_); 1718(*ν*_C=O_); 1660(*ν*_C=N_); 907(*ν*_M-N_); 1438, 1346, 1083, 728 (*ν*_Pc_); UV-Vis(DMF) *λ*_max_/nm: 274, 695; Anal. Cald. for C_36_H_16_ N_8_O_8_Zn: C, 57.33; H, 2.17; N, 14.90; Found: C, 57.01; H, 2.44; N, 15.46.

Zn(II)Pc(NO_2_)_4_—1.3614 g (35.4%)Yield; green solid; m.p. >300 °C; IR (KBr) *ν*max/cm^−1^: 3089(*ν*_C-H_); 1522, 1336(*ν*_N__-__O_); 1615(*ν*_C=N_); 916 (*ν*_M-N_); 1094, 737(*ν*_Pc_); UV–Vis (DMF) *λ*max/nm: 273, 674; Anal. Cald. For C_36_H_12_N_12_O_8_Zn: C, 53.65; H, 1.50; N, 20.86; Found: C, 52.05; H, 1.44; N, 20.33.

### 3.3. Evaluation of the Photocatalytic Activity

A 100 mL three-neck flask was selected for the oxidative desulfurization experiment; the oxygen cylinder and reflux condenser were linked to the flask. Catalyst powder (20 mg) was added to the model fuel system (100 mL, 800 μL·L^−1^) and oxygen with the rate of 100 mL·min^−1^ was bubbled into it. The experimental temperature of oxidative desulfurization was selected as 60 °C and carried out at standard atmospheric pressure [20]. The sample (2 mL) was collected every 30 min and put into the centrifuge to eliminate the catalyst particles. The residue sulfur concentration and the oxidation products of dibenzothiophene(DBT) in model fuel were analyzed by gas chromatography (Flame Photometric Detector, Agilent 6890 (Shanghai, China). The GC was equipped with a HP-5 capillary column (30 mm × 0.2 mm × 0.5 μm). The calculation formula of desulfurization rate of the model fuel was calculated by the equation given below (1):

(1) $$D(\%)\;=\;(C_{0}-C)/C_{0}\;\times 100\%$$

where *C*_0_ is the initial concentration, and *C* is the sulfur content concentration after a period of time.

## 4. Conclusions

In conclusion, a biomimetic catalytic system composed of metallophthalocyanines MPcR_4_ and molecular O_2_ was performed to study the significant influence of MN_4_-type coordination structure on the degradation of dibenzothiophene(DBT). The results reveal that the conjugate structures of phthalocyanines and the center metal ions played key roles in oxidative desulfurization performance. The inductive effect of different R substituents and the d electronic configuration strongly affected the electron cloud distribution of the conjugate structures and the catalytic performance of phthalocyanines. Additionally, mechanistic studies revealed that the MPcR_4_−O_2_·species were the main active intermediates. The results indicate that the skeleton structure of MPcR_4_ strongly affected the catalytic performance of phthalocyanines and encouraged us to design phthalocyanine compounds with different substituents.

## Figures and Tables

**Table 1:** HOMO orbital composition in phthalocyanine complex molecule [18,19].

M(II)Pc	HOMO		
	Orbital Type	Pc ring Component	M(II) d Orbital Composition
Mn(II)	σ	*s, p_x_ p_y_* (C,N)	*4s, 4p_x_ d_x2−y2_* (−0.0845)
Fe(II)	σ	*s, p_x_ p_y_* (C,N)	*4s, 4p_x_ d_x2−y2_* (0.1032)
Co(II)	π	*p_z_* (C,N)	*d_xz_, d_yz_* (0.0102)
Ni(II)	π	*p_z_* (C,N)	*d_xz_, d_yz_* (0.0509)
Cu(II)	π	*p_z_* (C,N)	*d_xz_, d_yz_* (0.0070)
Zn(II)	π	*p_z_* (C,N)	*d_xz_, d_yz_* (d10)

## Sample Availability

Not available.

## References

[1] Stanislaus A., Marafi A., Rana M.S. (2010). Recent advances in the science and technology of ultra low sulfur diesel (ULSD) production. Catal. Today.

[2] Sorokin A.B. (2013). Phthalocyanine Metal Complexes in Catalysis. Chem. Rev..

[3] Tailleur R.G., Ravigli J., Quenza S., Valencia N. (2015). Catalyst for ultra-low sulfur and aromatic diesel. Appl. Catal. A.

[4] Ma C., Dai B., Xu C., Liu P., Qi L. (2013). Deep oxidative desulfurization of model fuel via dielectric barrier discharge plasma oxidation using MnO_2_ catalysts and combination of ionic liquid extraction. Catal. Today.

[5] Fatima M., Luis D., Mariana S., Ribeiro S.O., Corvo M.C. (2018). Efficient heterogeneous polyoxometalate-hybrid catalysts for the oxidative desulfurization of fuels. Catal. Commun..

[6] Li S.W., Li Y.Y., Yang F., Liu Z., Gao R.M. (2015). Photocatalytic oxidation desulfurization of model diesel over phthalocyanine/La_0.8_Ce_0.2_NiO_3_. J. Colloid Interface Sci..

[7] Li Q., Wang H., Li Y., Li Y., Duan Q. (2018). Conjugated microporous polymers bearing metallophthalocyanine moieties with enhanced visible-light photocatalytic activity. Dye Pigment..

[8] Zhang G., Ren J., Liu B., Tian M., Zhou H. (2018). In situ hydrothermal preparation and photocatalytic desulfurization performance of metallophthalocyanine sensitized SnO_2_. Inorg. Chim. Acta.

[9] Xie J.F., Ma G.F., Ouyang X.P., Zhao L.S., Qiu X.Q. (2020). Metalloporphyrin as a Biomimetic Catalyst for the Catalytic Oxidative Degradation of Lignin to Produce Aromatic Monomers. Waste Biomass Valorization.

[10] Wang X.J., Shi Y.C., Zhuang S.G., Liang Z.X., Li B.T. (2019). Enhancement of Electricity Generation in Single Chamber Microbial Fuel Cell Using Binuclear-Cobalt-Phthalocyanine and Cerium Oxide Co-supported on Ordered Mesoporous Carbon as Cathode Catalyst. J. Electrochem. Soc..

[11] Zhu B.Q., Zhang X.J., Han M.L., Deng P.F., Li Q.L. (2015). Novel Planar Binuclear Zinc Phthalocyanine Sensitizer for Dye-Sensitized Solar Cells: Synthesis and Spectral, Electrochemical, and Photovoltaic Properties. J. Mol. Struct..

[12] Aykanat A., Meng Z., Benedetto G., Mirica K.A. (2020). Molecular Engineering of Multifunctional Metallophthalocyanine-Containing Framework Materials. Chem. Mater..

[13] Xu Z., Li K., Wang R., Duan X., Liu Q., Zhang R., Zhao J. (2017). Electrochemical Effects of Lithium-Thionyl Chloride Battery by Central Metal Ions of Phthalocyanines-Tetraacetamide Complexes. J. Electrochem. Soc..

[14] Koçyiğit N., Özen Ü.E., Özer M., Salih B., Özkaya A.R. (2017). Electrocatalytic Activity of Novel Ball-Type Metallophthalocyanines with Trifluoro Methyl Linkages in Oxygen Reduction Reaction and Application as Zn-Air Battery Cathode Catalyst. Electrochim. Acta.

[15] Yang Y., Tao X., Xu K., He H., Xu N. (2016). Effects of microwave/HAc-H_2_O_2_ desulfurization on properties of Gedui high-sulfur coal. Fuel Process. Technol..

[16] Arellano U., Shen J.M., Wang J.A., Timko M.T., Chen L.F. (2015). Dibenzothiophene oxidation in a model diesel fuel using CuO/GC catalysts and H_2_O_2_ in the presence of acetic acid under acidic condition. Fuel.

[17] Zhen Y., Li J., Wang D., Fu F., Xue G. (2015). Synthesis of α-MoO_3_ Nanobelt and Its Photocatalytic Oxidative Desulfurization (Photo-ODS) Activity of Simulation Fuel. J. Inorg. Mater..

[18] Schwenk C.F., Rode B.M. (2010). New insights into the Jahn-Teller effect through ab initio quantum-mechanical/molecular-mechanical molecular dynamics simulations of CuII in water. Chem. Phys. Chem..

[19] Liang Q., Zhang M., Liu C., Xu S., Li Z. (2016). Sulfur-doped graphitic carbon nitride decorated with zinc phthalocyanines towards highly stable and efficient photocatalysis. Appl. Catal. A.

[20] Zhou X., Li J., Wang X., Jin K., Ma W. (2009). Oxidative desulfurization of dibenzothiophene based on molecular oxygen and iron phthalocyanine. Fuel Process. Technol..

[21] Cynthia M.A., Wesley M.S., Johan E.V.L. (2001). urrent Status of Phthalocyanines in Photodynamic Therapy of Cancer. J. Porphyrin. Phthalocyanine.

[22] Huang J.L. (2001). Some Spectrum Methods on the Structures of Metal Phthalocyanine. Spectro. Spectral Anal..

[23] Gottfried J.M. (2015). Surface chemistry of porphyrins and phthalocyanines. Surf. Sci. Rep..

[24] Zhang X., Lin W., Zhao H., Wang R. (2018). Raman spectra study of p-tert-butylphenoxy-substituted phthalocyanines with different central metal and substitution positions. Vib. Spectrosc..

[25] Fu Q., Chen B., Zhao C., Zhang H., Du X., Shao Y., Zhao B. (1993). Study on the electron structure of binuclear metal phthalocynine. J. Northeast. Norm. Univ..

[26] Chen B., Du X., Yang S., Zhao C., Liu J. (1996). Study on the mechanisms of catalytic desulfurization with binuclear metal cations for the catalytic activity of MPc-PcM. J. Mol. Sci..

[27] Xu T., Ni D., Chen X., Wu F., Ge P. (2016). Self-floating graphitic carbon nitride/zinc phthalocyanine nanofibers for photocatalytic degradation of contaminants. J. Hazard. Mater..

[28] Lu W., Xu T., Wang Y., Hu H., Li N. (2016). Synergistic photocatalytic properties and mechanism of g-C_3_N_4_ coupled with zinc phthalocyanine catalyst under visible light irradiation. Appl. Catal. B.

[29] Zhang R., Wang J., Xu B., Huang X., Xu Z., Zhao J. (2012). Catalytic Activity of Binuclear Transition Metal Phthalocyanines in Electrolyte Operation of Lithium/Thionyl Chloride Battery. J. Electrochem. Soc..

[30] Cruz Moraes F., Cabral M.F., Machado S.A., Mascaro L.H. (2010). Electrocatalytic Behavior of Glassy Carbon Electrodes Modified with Multiwalled Carbon Nanotubes and Cobalt Phthalocyanine for Selective Analysis of Dopamine in Presence of Ascorbic Acid. Electroanalysis.

